# Bioactive fungal metabolites as SIRT2 antagonists: A computational quest for cancer treatment

**DOI:** 10.1371/journal.pone.0339474

**Published:** 2025-12-22

**Authors:** Md. Habib Ullah Masum, Syed Mohammad Lokman, Kazi Chamonara, Md Shahidur Rahman, Erfanul Haq Chowdhury, Rehana Parvin, Md. Rimon Bhuiyan, Md Nafis Enam, Ahmad Abdullah Mahdeen

**Affiliations:** 1 Department of Genomics and Bioinformatics, Faculty of Biotechnology and Genetic Engineering, Chattogram Veterinary and Animal Sciences University, Khulshi, Bangladesh; 2 Asian University for Women (AUW), Chattogram, Bangladesh; 3 Department of Environmental Biotechnology, Faculty of Biotechnology and Genetic Engineering, Chattogram Veterinary and Animal Sciences University, Khulshi, Bangladesh; 4 Department of Microbiology, Noakhali Science and Technology University, Noakhali, Bangladesh; 5 Lovely Professional University, Phagwāra, Punjab, India; 6 Department of Pathology and Parasitology, Faculty of Veterinary Medicine, Chattogram Veterinary and Animal Sciences University, Khulshi, Bangladesh; 7 Department of Veterinary and Animal Sciences, University of Rajshahi, Rajshahi, Bangladesh; University of Texas Southwestern, UNITED STATES OF AMERICA

## Abstract

SIRT2, a member of the sirtuin protein family, plays a pivotal role in regulating tumor progression by modulating key metabolic pathways and signaling proteins. The regulatory role of this protein has been documented across a variety of cancers. Emphasizing its role in cancer biology, this study employed computer aided drug design (CADD) approach, including molecular docking, dynamics and pharmacoinformatics, to screen fungal metabolites as potential anticancer agents. Subsequent assessments of pharmacokinetics and toxicity revealed that all tested fungal metabolites possessed oral bioavailability, drug-like characteristics, and favorable ADMET profiles. The metabolites also exhibited no hepatotoxicity, carcinogenicity, or mutagenicity, highlighting their significant therapeutic potential and favorable safety profile. The docking analysis further revealed strong binding affinities of the metabolites with the SIRT2, with the MSID001658 showing the highest score (–10.9 kcal/mol), followed by the MSID000672 (−10.2 kcal/mol). Further molecular dynamics simulation evaluated the structural and dynamic stabilities of the SIRT2 in association with the ligands. Although both complexes showed overall stability, the MSID000672 had larger RMSD variations, whereas the MSID001658 maintained consistent structural integrity throughout the simulation. Furthermore, the binding of MSID000672 was associated with increased solvent exposure, in contrast to the more compact molecular surface area (MolSA) and radius of gyration (Rg) profile observed for the MSID001658. The PCA indicated compact clustering (53.6%) for the SIRT2_MSID000672 complex, whereas the SIRT2_MSID001658 had a larger variance (70.9%) of flexibility. The DCCM further revealed enhanced coordination of internal movements in the SIRT2_MSID000672 complexes. Finally, the MSID001658 fosters a compact, stable complex with the SIRT2, whereas the MSID000672 increases conformational flexibility and solvent accessibility. These results support the notion that the MSID000672 might be an effective anticancer agent if subjected to more experimental trials.

## 1. Introduction

Recent global projections from GLOBOCAN 2022 indicate roughly 20 million new cancer diagnoses and 9.7 million cancer-related fatalities globally [1]. Lung cancer continues to be the most prevalent cause of cancer-related mortality, accounting for 18.7% of all cancer deaths [1]. Following lung cancer, the most significant contributors to global cancer mortality were colorectal cancer (9.3%), liver cancer (7.8%), female breast cancer (6.9%), and stomach cancer (6.8%) [1]. Geographically, Asia comprises about 49% of new cancer cases and over 56% of cancer fatalities. Despite incidence and death, cancer prevalence is significant, with about 53.5 million individuals surviving five years post-cancer diagnosis in 2022 [2].

Sirtuins (SIRTs) represent a distinct class of histone deacetylases, commonly referred to as class III histone deacetylases (HDACs), which are unique in their dependency on nicotinamide adenine dinucleotide (NAD⁺) as a cofactor for protein deacetylation [3–5]. Recent research has demonstrated that sirtuins are capable of catalyzing a variety of post-translational modifications, such as desuccinylation and demyristoylation, along with deacetylation [6,7]. The mammalian genome encodes seven distinct members of sirtuins (SIRT1−7), each characterized by a specific subcellular localization [8]. From these SIRTs, SIRT6 and SIRT7 are predominantly localized within the nucleus, whereas SIRT3 to SIRT5 are confined to the mitochondria. In contrast, SIRT1 and SIRT2 are distributed across both the cytoplasm and the nucleus [9]. The substrates of SIRT2 can include both histone and non-histone types, encompassing a variety of enzymes associated with the cell cycle, metabolic processes, transcription factors, substrates linked to cell signaling, and structural proteins [10–13]. The SIRT2 is found in multiple organs such as the brain, ovary, esophagus, heart, liver, lung, testicles, thyroid, and spleen. Many studies have shown that the SIRT2 plays a significant role in regulating tumor progression through the modulation of various substrates involved in metabolic and signaling pathways [14]. This regulatory function of the SIRT2 is evident across multiple cancer types, including glioma [15,16], liver [17,18], lung [19,20], gastric [21], and colon cancers [22]. In glioma, the SIRT2 deacetylates G6PD, enhancing NADPH and pentose phosphate production, which supports the metabolic needs of rapidly proliferating tumor cells [15]. Additionally, deacetylation of P73 by the SIRT2 enhances the transactivation of PUMA, promoting apoptosis [16]. In liver cancer, the SIRT2 influences tumor progression through AKT activation, leading to GSK-3 inhibition and elevated β-catenin levels, which drive metastasis [23]. Concurrently, the SIRT2-mediated activation of phosphoenolpyruvate carboxykinase 1 (PEPCK1) promotes glycolysis and reduces E-cadherin expression, facilitating tumor cell invasion [24]. In lung cancer, the SIRT2 targets P53 and pyruvate dehydrogenase E1 subunit alpha 1 (PDHA). This deacetylation of P53 leads to reduced apoptosis in non-small cell lung cancer (NSCLC) cells, thereby supporting tumor survival. Simultaneously, PDHA1 activation enhances glycolytic metabolism, a hallmark of cancer cell energetics [19,20]. In gastric cancer, the SIRT2 modulates PEPCK1, which in turn activates the RAS/ERK/JNK/MMP-9 pathway, promoting metastasis and tissue invasion [21]. Moreover, colon cancer progression is influenced by the SIRT2 through its regulation of NF-κB and CSN2, triggering the NF-κB/CSN2 signaling axis that induces Snail expression and enhances metastatic potential [22].

The extensive array of the SIRT2 substrates indicates their participation in several biological processes [25,26]. Thus, irregular SIRT2 activity has been associated with the onset and dissemination of cancer diseases [27,28]. The substrate is therefore an emerging pharmacological target for therapeutic intervention [12]. Also, this substrate, similar to other sirtuins, has two structural domains: the Rossmann fold domain (RFD) and the zinc-binding domain (ZBD) [29,30]. The binding site of SIRT2 is located inside a broad hydrophobic groove between the two domains. However, the active site of this substrate has an acetyl-lysine channel, several hydrophobic pockets, and a ligand-induced selectivity pocket [31,32]. Sirreal2 is one of the few confined SIRT2 inhibitors found in the hydrophobic pocket subsequent to the ZBD [32]. Where the dimethyl mercaptopyrimidine moiety provides a binding and selectivity pocket, the naphthyl group extends into the acetyl-lysine channel, causing van der Waals interactions with nicotinamide [33].

Computer-aided drug design (CADD) approach for discovering novel drugs has garnered significant interest because of its potential to accelerate the discovery process in terms of time, labor, and costs [34]. This approach has been employed to develop a wide array of novel medications successfully. Traditionally, a significant number of innovative treatments have been derived from natural sources as secondary metabolites [35]. Recently, medicinal fungi and plant metabolites have gained attention as a valuable source of bioactive secondary metabolites that may offer therapeutic benefits for various complex diseases [36–38]. Therefore, the study employed a CADD approach, including molecular docking and pharmacoinformatic approaches, to identify and screen fungal metabolites derived from fungal medicinal plants as potential therapeutic agents against SIRT2. The study aimed to analyze the interaction patterns of these compounds with key therapeutic targets, providing insights into their potential efficacy in combating the disease.

## 2. Methodology

### 2.1. Retrieval of fungal metabolites and receptor protein

In this study, fungal metabolites were identified and retrieved from the Medicinal Fungi Secondary Metabolite And Therapeutics (MeFSAT) (https://cb.imsc.res.in/mefsat/) [39]. From the database, a total of 1830 secondary metabolites were identified from 184 medicinal fungi; each chosen for possible therapeutic potential against SIRT2. The chemical structures of the fungal metabolites were obtained in PDB format from the server database to prepare the ligand. These structures were further optimized and visualized by PyMOL (https://www.pymol.org/) software. Since no complete tertiary structures of the SIRT2 protein are available in the Protein Data Bank (PDB), the predicted tertiary structure was retrieved from the AlphaFold database (AF ID: AF-Q8IXJ6-F1-v4; UniProt ID: Q8IXJ6). The obtained model demonstrated high confidence, with an average pLDDT score of 81.84. Before molecular docking, the protein structure underwent refinement via GalaxyRefine (https://galaxy.seoklab.org/cgi-bin/submit.cgi?type=REFINE) [40] and was further optimized using AutoDock Tools (http://vina.scripps.edu/) to enhance docking accuracy [41]. Subsequently, the structural validation of the vaccine models was performed using the SAVES v6.0 server (https://saves.mbi.ucla.edu/), which evaluates the stereochemical quality of the predicted structures through Ramachandran plot and ERRAT analysis [42–45].

### 2.2. Molecular docking analysis

Molecular docking is a fundamental technique in structure-based drug design, commonly used in computer-aided drug design (CADD) to predict optimal binding conformations of micromolecules (ligands) to target macromolecules (receptor protein) [46]. In this study, site-specific docking was performed to identify potential binding sites on the receptor protein (SIRT2) by using the PyRx (https://pyrx.sourceforge.io/) software [47]. During the docking analysis, the grid box was adjusted according to the active sites of SIRT2, identified from the available ligand-bound PDB structure (PDB ID: 4RMG). As a control SIRT2 inhibitor, the binding sites of the sirreal and AGK2 were applied for subsequent site-specific docking analysis. The resultant grid box parameters, such as grid centre (X:Y:X = 4.57:1.44:-3.44) and size (X:Y:Z = 87.54:125.72:144.98), were applied for the rest of the assessed fungal metabolites. The subsequent docking analysis proceeded with an exhaustiveness value of 24. The receptor-ligand interacting residues were further visualized and analyzed with BIOVIA Discovery Studio Visualizer [48].

### 2.3. Biophysical, pharmacokinetic, and drug-likeness properties

The biophysical, pharmacokinetic, and drug-likeness properties of the fungal secondary metabolites, including absorption, distribution, metabolism, and excretion (ADME), were evaluated using SwissADME http://www.swissadme.ch/index.php) server. Following molecular docking, the SMILES notations were retrieved from the PubChem database (https://pubchem.ncbi.nlm.nih.gov/) and subjected to physicochemical, pharmacokinetic, and drug-likeness properties assessment using the SwissADME (http://www.swissadme.ch/index.php) [46]. The server predicts key descriptors, including lipophilicity, solubility, gastrointestinal absorption, blood-brain barrier permeability, drug-likeness, and Lipinski’s Rule of Five. Additionally, it evaluates interactions with cytochrome P450 enzymes and identifies potential issues such as low solubility or bioavailability [46,49].

Early toxicity prediction proves crucial for optimizing lead compounds and reducing failure risks in drug development [50]. The potential toxicity of the selected fungal metabolites was evaluated by using the ProTox 3.0 server (https://tox.charite.de/protox3/), which predicts the toxicological profiles of bio-compounds using computational algorithms and extensive toxicological datasets. It provides predictions for various toxicity endpoints, including oral toxicity, hepatotoxicity, and carcinogenicity, and categorizes compounds into six toxicity classifications [51].

### 2.4. Molecular dynamics simulation and post-simulation analysis

Molecular Dynamics (MD) simulation serves as a robust computational methodology used to investigate the chemical and physical behaviors of dynamic biomolecular systems, such as protein-ligand (P-L) complexes or receptor structures [52]. This methodology enables researchers to investigate various molecular phenomena, including comprehensive protein profiling, identification of novel binding sites, and assessment of thermodynamic characteristics such as free energy. In this study, MD simulations were specifically employed to analyze further the top two docked ligand candidates. Among these, compounds identified in the MeFSAT database as MSID000672 and MSID001658 were selected for an in-depth dynamic evaluation over a 100-nanosecond (ns) simulation, focusing on their interaction with the SIRT2 protein. The simulations were carried out using the Desmond module, which is part of the Schrödinger software suite.

To emulate realistic biological conditions and maintain volumetric consistency, each protein-ligand complex was immersed within an orthorhombic simulation box filled with TIP4P water molecules, with the dimensions set to 10 × 10 × 10 Å^3^. Sodium (Na⁺) and chloride (Cl⁻) ions were included at a concentration of 0.15 M to replicate physiological ionic strength, with their distribution being random throughout the solvated system. The OPLS3e force field was employed to optimize the system’s energy landscape, offering a reliable framework for simulating interatomic interactions [53]. The steepest descent method was used during this optimization, ensuring an effective minimization of system energy. Equilibration steps were subsequently performed under the NPT ensemble, maintaining a constant pressure of 1.01325 bar and a stable temperature of 300 K. Trajectory data were recorded at intervals of 100 picoseconds (ps), allowing for detailed tracking of the system’s evolution over time [54].

This simulation approach enables the emulation of contemporaneous molecular dynamics, monitoring structural modifications of protein-ligand complexes over the simulation period. Several analytical parameters were evaluated to assess system stability and interaction dynamics. These analyses encompassed root mean square fluctuation (RMSF) and root mean square deviation (RMSD), which served to elucidate residue-level mobility and overall structural stability, respectively. Further assessments encompassed the analysis of hydrogen bonds, solvent accessible surface area (SASA), molecular surface area (MolSA), polar surface area (PSA), and radius of gyration (Rg). The Simulation Interaction Diagram (SID) tool from Schrödinger was used to investigate P-L and ligand-protein (L-P) interactions further. To improve comprehension of the conformational changes and associated motions within the biomolecular system, analyses utilizing Principal Component Analysis (PCA) and Dynamic Cross-Correlation Matrix (DCCM) were conducted. The implementation of the above assignments was carried out using the Bio3D package in the R programming environment. These computational tools are essential for expediting the discovery of potential medication candidates by predicting the affinity and stability of small compounds interacting with target proteins.

## 3. Results

**Fig 1:**
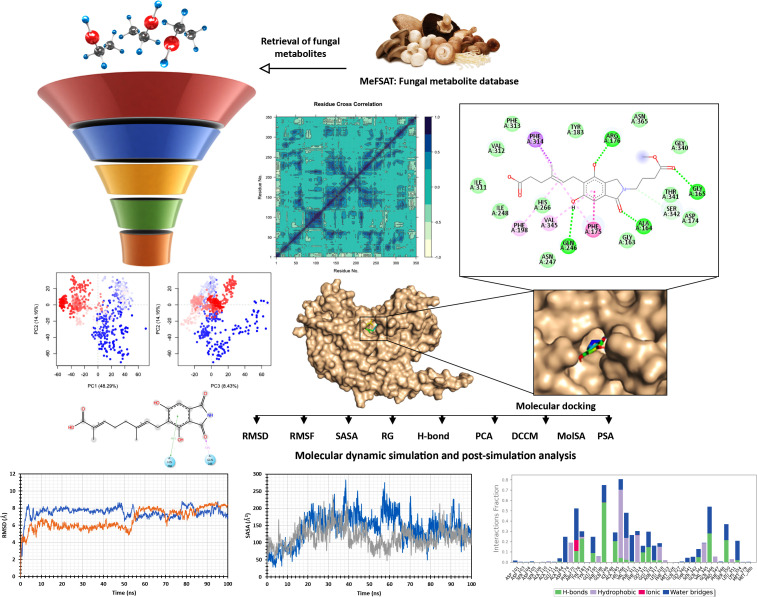
The overview of the entire study.

The overview of the study is depicted in Fig 1.

### 3.1. Molecular docking analysis

**Fig 2:**
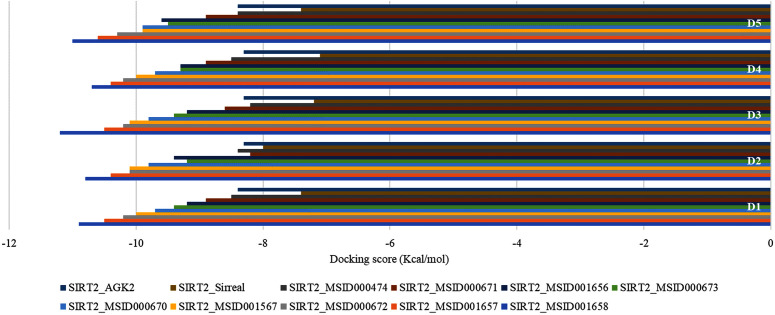
The docking scores of the SIRT2-ligand complexes. Five consecutive docking analyses are presented as D1-D5.

A total of 1,830 fungal secondary metabolites were extracted from the MeFSAT database, with their tertiary structures obtained in PDB format and produced as ligands. The refined 3D model of the receptor SIRT2 protein was later obtained from the GalaxyWEB server, with an RMSD of 0.486, a MolProbity score of 1.493, and 98.7% of residues in Ramachandran’s favored region. The SAVES Ramachandran plot showed that 94.9% of amino acid residues in the refined receptor protein were located in the most favored region, with 4.8% in the additionally and generously allowed regions, while the ERRAT score of the model was 93.17. Prior to molecular docking, the SIRT2 protein structure was optimized by eliminating water molecules, metal ions, and cofactors, including polar and nonpolar hydrogens, and computing Gasteiger charges. The final protein structures were prepared in PDBQT format using AutoDock Tools, and docking analyses were conducted with PyRx. A site-specific docking method was subsequently utilized to pinpoint the potential binding sites of the receptor protein SIRT2 using the binding sites of the sirreal and AGK2. These control substances exhibited docking scores of −7.4 kcal/mol and −8.4 kcal/mol, respectively. Subsequent docking analyses at the binding sites revealed that the binding affinities of all tested fungal secondary metabolites varied from −7.0 to −10.9 kcal/mol. According to their docking scores, nine fungal metabolites, including MeFSAT ID MSID001658, MSID001657, MSID000672, MSID001567, MSID000670, MSID000673, MSID001656, MSID000671, and MSID000474, exhibiting binding affinities of ≤ −8.5 kcal/mol, were identified as having strong interactions and were chosen for additional virtual screening and analyses (S1 Table, Fig 2).

### 3.2. Biophysical, pharmacokinetic, and drug-likeness properties

The biophysical profiles of nine fungal metabolites were evaluated using SwissADME, demonstrating varied molecular properties. The molecular weights varied from 290.27 g/mol (MSID000474) to 435.51 g/mol (MSID000670), with heavy atom counts ranging from 21 to 32. Aromatic heavy atoms were mostly homogeneous throughout the compounds, with most containing six, except for MSID000670 and MSID000474, which had twelve. The proportion of sp^3^-hybridized carbons (Fraction Csp^3^), reflecting molecular complexity, varied from 0.2 (MSID000474) to 0.53 (MSID001658 and MSID001657). The molar refractivity values, indicative of molecule polarizability, ranged from 74.33 to 128.69, although the counts of rotatable bonds exhibited significant variation, from one in MSID000474 to ten in MSID001567 and MSID001656, highlighting disparities in molecular flexibility. The topological polar surface area (TPSA), significant for forecasting drug transport characteristics, varied from 95.86 Å^2^ (MSID000671) to 124.37 Å^2^ (MSID001567 and MSID001656) (S2 Table). The observations underscore a wide range of structural variation among fungal metabolites, potentially affecting their pharmacokinetic properties.

The SwissADME prediction for the pharmacokinetics of nine fungal metabolites showed consistently high gastrointestinal absorption (GI) for all compounds, indicating promising oral bioavailability. None of the metabolites was expected to cross the blood-brain barrier (BBB). Among the nine metabolites, six metabolites, such as MSID001658, MSID001657, MSID001567, MSID001656, MSID000671, and MSID000474, have been recognized as substrates for P-glycoprotein (P-gp), potentially affecting their efflux and bioavailability. Selective interactions were noted regarding the inhibition of cytochrome P450 enzymes: MSID000672, MSID001567, MSID000673, MSID001656, and MSID000671 were found to inhibit CYP1A2; MSID000670 specifically inhibited CYP2C9, while CYP2D6 and CYP3A4 showed no inhibition. Interestingly, none of the metabolites demonstrated broad-spectrum inhibition across various CYP isoforms. The anticipated values for skin permeability (Log Kp) varied from –5.63 cm/s (MSID000670) to –8.07 cm/s (MSID001658 and MSID001657) (S3 Table).

All nine metabolites adhered completely to Lipinski’s rule of five. Furthermore, each chemical satisfied the requirements established by the Ghose, Veber, Egan, and Muegge filters. The bioavailability scores for the metabolites were consistently elevated, ranging from 0.55 to 0.56 (S4 Table). The Protox 3.0 predicted toxicity profiles of chosen fungal metabolites across six key toxicity endpoints: hepatotoxicity, carcinogenicity, mutagenicity, cytotoxicity, cardiotoxicity, and nephrotoxicity. The compounds largely exhibited a remarkable safety profile, being deemed inactive across all evaluated toxicity parameters. Among the metabolites examined, only MSID001657 exhibited potential mutagenic activity, while it remained inactive in all other categories. The findings indicate that these fungal metabolites have low toxicity risks, highlighting their promise as safe options for future pharmacological development (S5 Table).

### 3.3. Post-docking analysis

**Fig 3:**
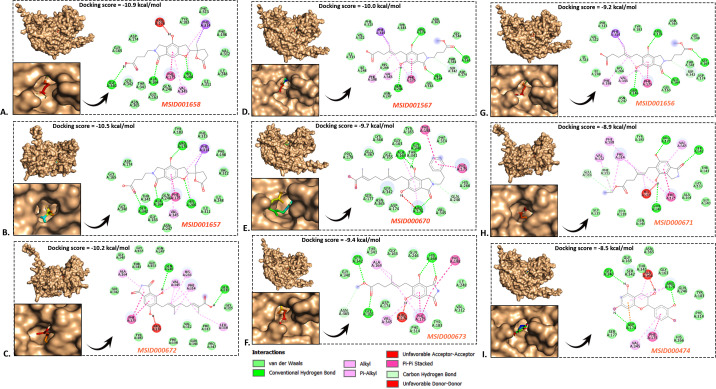
Post-docking analysis of the SIRT2-ligand complexes. The intermolecular interactions, including van dar Waals, conventional hydrogen, alkyl or π -alkyl, π- π, and unfavorable bonds are depicted in cyan, green, light pink, dark pink and red colors, respectively.

The post-docking evaluation of fungal metabolites MSID001658, MSID001657, MSID000672, MSID001567, MSID000670, MSID000673, MSID001656, MSID000671, and MSID000474 with SIRT2 demonstrated significant binding affinities. The compound MSID001658 exhibited the most robust binding, achieving a docking score of −10.9 kcal/mol. It was closely followed by MSID001657 and MSID000672, which recorded scores of −10.5 and −10.2 kcal/mol, respectively. The three compounds demonstrated the strongest binding affinity. MSID001567 and MSID000670 demonstrated strong binding, achieving docking scores of −10.0 and −9.7 kcal/mol, respectively. The binding affinities for MSID000673, MSID001656, and MSID000671 were moderate, falling within the range of −9.4 to −8.9 kcal/mol. The lowest binding strength was noted for MSID000474, showing a docking score of −8.5 kcal/mol (Fig 3).

The P-L complexes exhibited diverse intermolecular interactions, including van der Waals forces, conventional bonds, and carbon-hydrogen bonds. In the SIRT2_MSID001658, the ligand established many conventional hydrogen bonds with residues, including ALA:164, HIS:266, GLY:340, and particularly with ARG:176. Multiple van der Waals interactions were identified with residues such as GLY:163, GLY:165, ASN:365, ILE:311, and ILE:248. The ligand reported π–π stacking interactions with PHE:175 and PHE:314, as well as π–alkyl interactions with VAL:345, PHE:175, and PHE:314. Furthermore, only one unfavorable donor–donor interaction was seen with ARG:176 (Fig 3A). The ligand in SIRT2_MSID001657 established conventional hydrogen bonds with several important residues, such as ALA:164, GLN:246, HIS:266, SER:342, and THR:341. A further hydrogen bond was observed with ARG:176. Interactions of the van der Waals were observed with several nearby residues, including GLY:163, GLY:165, ASN:247, ILE:311, ILE:248, VAL:312, and TYR:183. Additionally, the ligand was reported to participate in π–π stacking interactions with PHE:175, as well as π–alkyl interactions with VAL:345. A π–sigma interaction was noted with PHE:314 (Fig 3B).

In the SIRT2_MSID000672, the ligand established many conventional hydrogen bonds with the residues GLN:246 and GLU:316. An unfavorable donor–donor interaction was noted with ARG:176. A considerable quantity of π–π stacking contacts was formed with aromatic residues such as PHE:175, PHE:314, and PHE:313. Interactions involving π–alkyl and alkyl groups were observed with VAL:345, VAL:312, and LEU:318. A π–sigma contact was established with ALA:164, contributing to the non-covalent aromatic interactions. Multiple van der Waals contacts were observed with adjacent residues, including GLY:165, THR:341, ASN:247, GLY:163, TYR:183, PHE:198, GLN:346, and PRO:347 (Fig 3C). The ligand within SIRT2_MSID001567 established multiple conventional hydrogen bonds with the residues ALA:164, GLY:165, ARG:176, and GLN:246. A variety of van der Waals interactions were observed with nearby residues, including ASN:247, HIS:266, ILE:248, ILE:311, VAL:312, PHE:313, TYR:183, ASN:365, GLY:340, THR:341, and ASP:174. A π–-sigma interaction was established with PHE:175, while a carbon-hydrogen interaction was formed with SER:342. Interactions involving π–alkyl and alkyl groups were observed with PHE:198 and VAL:345 (Fig 3D). The ligand demonstrated several important interactions within SIRT2_MSID000670, including carbon-hydrogen bonds with GLN:246 and HIS:266, as well as conventional hydrogen bonds with residues such as THR:341 and ARG:176. Notably, the ARG:176 exhibited negative donor-donor interactions. There were several van der Waals interactions with residues such as ASP:174, SER:177, GLY:165, and ALA:164. Together with π–alkyl interactions and π–π stacking interactions with aromatic residues like PHE:198 and PHE:175, these interactions further stabilized the ligand within the binding pocket (Fig 3E).

In SIRT2_MSID000673, conventional hydrogen bonds were identified with residues SER:342, GLY:165, and HIS:266, while carbon-hydrogen bonding existed with ASN:365. An unfavored donor–donor interaction was observed between the ligand and ARG:176. Van der Waals interactions were prevalent, including residues such as ALA:164, ASP:174, VAL:345, and TYR:183. Moreover, substantial π-contacts were observed, including π–π stacking and π–π interactions with PHE:175 and PHE:198, as well as π–alkyl interactions with ALA:164 and PHE:314 (Fig 3F). In SIRT2_MSID001656, conventional hydrogen bonds were established with key residues such as ARG:176, GLN:246, and PHE:175. The presence of carbon-hydrogen bonds with residues such as THR:341, GLY:165, and ALA:164 contributed to the overall structural stability of the protein. A broad range of van der Waals interactions were seen with residues such as HIS:266, GLY:163, ASP:174, and ASN:247. Interactions involving π played a significant role, particularly through stacking and σ interactions with aromatic residues, such as PHE:314, PHE:198, and PHE:175. Additionally, there were interactions with VAL:345 and HIS:266 that enhanced hydrophobic and electron cloud contacts (Fig 3G).

Within the SIRT2_MSID000671 complex, conventional hydrogen bonds were formed with key amino acids, including ARG:176, SER:342, and GLN:246. There was also evidence of carbon-hydrogen bonding, especially with GLU:316. A variety of van der Waals interactions were observed among residues, including THR:341, GLY:163, LEU:318, and ASN:346. Prominent interactions were reported with π–π stacking with aromatic residues like PHE:175, PHE:198, and PHE:314, as well as π–alkyl and alkyl interactions involving VAL:345 and PHE:313. Moreover, there was an unfavorable interaction between the acceptors of the ligand and HIS:266 (**Fig 3H**). In SIRT2_MSID000474, conventional hydrogen bonds were notably established with residues including ARG:176, THR:341, ASP:174, and GLY:165. A π-anion interaction was identified with ASP:174. Van der Waals interactions were prevalent, including residues such as SER:342, GLY:340, ASN:365, and HIS:266. π–π stacking and π–alkyl interactions with aromatic residues, including PHE A:175 and VAL A:345, further reported the nonpolar contacts and stabilized the ligand. Furthermore, a detrimental donor–donor interaction was seen with ALA:164 (**Fig 3I**).

In the SIRT2_sirreal, there were two notable π-anion interactions identified with GLU:264 and GLU:316. A typical hydrogen bond was established with ASP:310. The presence of carbon-hydrogen bonds between GLY:267 and LYS:308 contributed to improved molecular recognition by providing nuanced electrostatic complementarity. Interactions involving pi-alkyl with VAL:263 and VAL:312, as well as alkyl interactions with LEU:262 and PRO:319, were also observed. A pi-sigma interaction was observed with ARG:321. Furthermore, extensive van der Waals interactions with residues such as ASN:317, ILE:311, ARG:280, and THR:268, among others, contributed to structural compatibility and improved binding affinity (S1A Fig). Furthermore, in the SIRT2_AGK2, a significant π-cation interaction was noted with ARG:280. Furthermore, there were multiple π-alkyl interactions with residues, including PRO:319, ARG:321, and THR:268, as well as alkyl interactions with ILE:275, VAL:312, and LYS:308. A variety of van der Waals interactions with nearby residues, such as GLU:316, GLY:267, LEU:262, and PRO:259, among others, contributed to the stability and fitting of the ligand. However, no hydrogen bonds or negative interactions were observed (S1B Fig). Based on the favorable biophysical characteristics, pharmacokinetic properties, drug-likeness parameters, and docking analyses, the MSID000672 and MSID001658 were further subjected to detailed molecular dynamics simulation analysis to assess their molecular behavior.

### 3.4. Post-simulation analysis

#### 3.4.1. RMSD.

**Fig 4:**
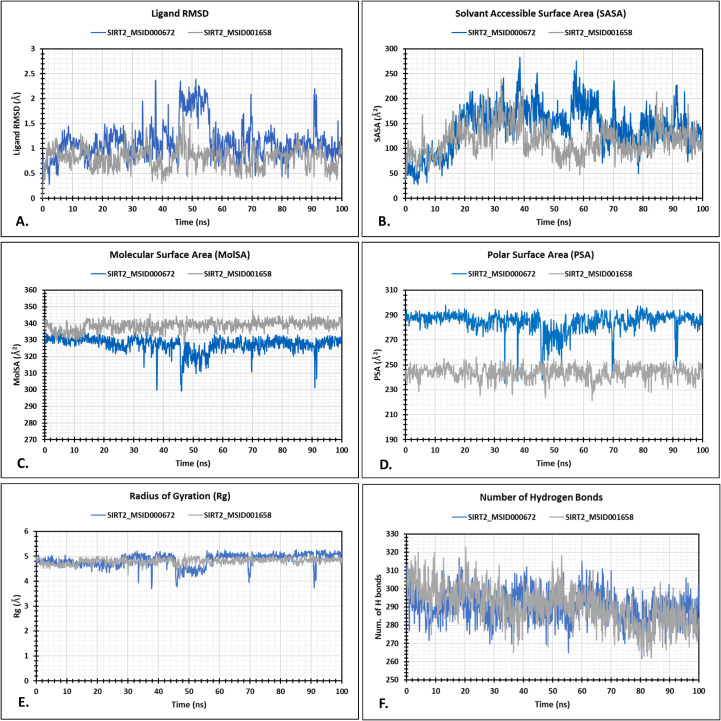
Post-simulation analysis of the protein-ligand complexes. The mean RMSD (A), SASA (B), MolSA (C), and PSA (D), Rg (E), and hydrogen bond (F) analysis of the ligand and protein-ligand complexes.

During a 100 ns simulation period, the MSID000672 and MSID001658 within the SIRT2_MSID000672 and SIRT2_MSID001658 complexes displayed notable trends. The RMSD quantifies the extent to which a set of atoms deviates from the precise reference structure of a protein, ligand, or ligand-protein complex. High RMSD values indicate a considerable degree of instability resulting from alterations in the structure of the molecule under investigation. The RMSD values for the SIRT2 complexes MSID000672 and MSID001658 were estimated to be 1.135 ± 0.370 Å and 0.808 ± 0.204 Å, respectively (Fig 4A; Table 1). The MSID000672 complex showed significant RMSD fluctuations at specific time intervals: 33–56 ns, 67–70 ns, and 91–93 ns. Conversely, the MSID001658 complex exhibited a slight rise in RMSD from 44 to 48 ns (Fig 4A).

**Table 1:** The mean RMSD, RMSF, RG, SASA, MolSA, and PSA analysis of the protein and protein-ligand complexes.

Docked complex		Mean RMSD ± SD Å	Mean RMSF ± SD Å	Mean Rg ± SD Å	Mean SASA ± SD Å²	Mean MolSA ± SD Å²	Mean PSA ± SD Å²
Ligand	MSID000672	1.135 ± 0.370 Å	–	–	–	–	–
	MSID001658	0.808 ± 0.204	–	–	–	–	–
Protein Cα	MSID000672	7.491 ± 0.729	1.918 ± 1.455	4.846 ± 0.258	141.815 ± 44.145	327.261 ± 4.582	284.158 ± 8.005
	MSID001658	6.673 ± 1.161	1.829 ± 1.001	4.799 ± 0.123	116.319 ± 30.000	338.616 ± 2.953	243.722 ± 5.259

Nonetheless, the SIRT2 showed notable differences between SIRT2_MSID000672 and SIRT2_MSID001658, as indicated by the RMSD values of 7.491 ± 0.729 Å and 6.673 ± 1.161 Å, respectively (Table 1). The comparative examination of protein dynamics revealed that the SIRT2_MSID001658 complex exhibited a more pronounced fluctuation pattern than the SIRT2_MSID000672 complex. In both instances, the SIRT2 protein exhibited significant variations beyond the 50 ns threshold. Notably, despite these mid-simulation fluctuations, RMSD values progressively decreased towards the end of the simulation (S2A Fig).

#### 3.4.2. RMSF.

The SIRT2 protein showed notable patterns in the SIRT2_MSID000672 and SIRT2_MSID001658 complexes over a simulation period of 100 ns. The protein exhibited RMSF values of 1.918 ± 1.455 Å and 1.829 ± 1.001 Å in the SIRT2_MSID000672 and SIRT2_MSID001658 complexes, respectively. The SIRT2_MSID000672 complex had considerably more protein fluctuations throughout the simulation, with marked areas of enhanced mobility identified at the residue ranges of 15–36, 66–78, 240–293, and 333–350 (**Table 1**, S2B Fig).

#### 3.4.3. SASA, MolSA, and PSA.

SASA is a critical parameter for understanding the structural stability and dynamic behavior of protein-ligand complexes. Over the 100 ns molecular dynamics simulation, the SIRT2-bound complexes of MSID000672 and MSID001658 exhibited distinct SASA profiles. The average SASA values were calculated to be 141.815 ± 44.145 Å^2^ for SIRT2_MSID000672 and 116.319 ± 30.000 Å^2^ for SIRT2_MSID001658 (Table 1; Fig 4B). Additionally, the MolSA and PSA were analyzed to assess their structural properties further. The average MolSA values were 327.261 ± 4.582 Å^2^ for SIRT2_MSID000672 and 338.616 ± 2.953 Å^2^ for SIRT2_MSID001658 (Table 1; Fig 4C). In terms of PSA, SIRT2_MSID000672 and SIRT2_MSID001658 exhibited average values of 284.158 ± 8.005 Å^2^ and 243.722 ± 5.259 Å^2^, respectively (Table 1; Fig 4D).

#### 3.4.4. Rg and hydrogen bond.

In a 100 ns simulation period, MSID000672 and MSID001658 displayed divergent structural patterns in association with SIRT2. The mean Rg values for these complexes were documented as 4.846 ± 0.258 Å and 4.799 ± 0.123 Å, respectively (Table 1; Fig 4F). Throughout a 100-ns simulation period, the SIRT2_MSID000672 and SIRT2_MSID001658 exhibited changes in hydrogen counts. The average values for hydrogen bonds in these compounds were determined as 290.293 ± 8.312 Å and 290.791 ± 10.306 Å, respectively (Table 1; Fig 4F).

#### 3.4.5. Protein ligand and ligand-protein contact.

**Fig 5:**
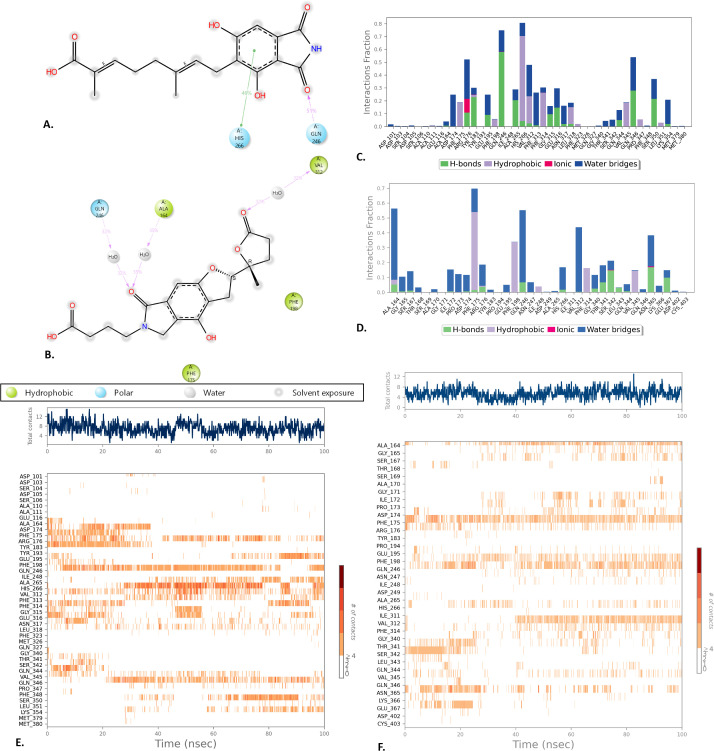
The intermolecular Protein-ligand interactions of the complexes during the simulation period. The ligand-protein (A, B) and protein-ligand (C, D) interactions of the SIRT2_MSID000672 and SIRT2_MSID001658 are depicted in a 2D structure diagram and a bar plot, respectively, where the heatmap (E, F) demonstrate the residual interactions based on simulation time frame.

In a 100 ns simulation time frame, the complex structure of a protein, together with its associated ligands and their intermolecular interactions, was analyzed using the SID. Various properties, including hydrogen bonds, ionic bonds, hydrophobic contacts, and water bridge bonds, were used to assess and demonstrate the interactions of ligand-protein and protein-ligand complexes. The MSID000672 demonstrated various functional groups, such as hydroxyl, carboxylic acid, and amide groups, along with a conjugated hydrocarbon chain and a substituted aromatic ring. Interactions with the ligand were facilitated by two amino acid residues from the protein—HIS:266 and GLN:246. The HIS:266 was associated with a hydroxyl-substituted aromatic ring, showing a 46% interaction probability, whereas GLN:246 exhibits a 51% interaction with a carbonyl oxygen on the ligand (Fig 5A**).** Conversely, the MSID001658 exhibited a fused aromatic ring system with many functional groups, including hydroxyls, carbonyls, esters, and a nitrogen-containing heterocycle. The water molecules facilitated interactions between the ligand’s carbonyl group and the residues GLN:246 and ALA:164, with interaction probabilities of 32% and 35%, respectively. A different water molecule facilitated a 32% contact with VAL 312 via the ester carbonyl group. Adjacent hydrophobic residues, including PHE:175, PHE:198, and VAL:312, presumably facilitated the stability of the ligand via hydrophobic or π-π interactions (Fig 5B).

The bar graph illustrates the types and frequencies of molecular interactions between a ligand and various amino acid residues of a protein, expressed as interaction fractions. The x-axis lists the residues by their names and sequence positions, while the y-axis shows the fraction of simulation time each interaction type occurs. Four types of interactions are represented: hydrogen bonds, hydrophobic contacts, ionic interactions, and water bridges. In the SIRT2_MSID000672, significant findings included robust and persistent water bridge interactions, particularly with residues PHE:175, GLN:246, GLN:346, VAL:312, and PHE:313. Hydrogen bonding was especially significant with GLN:246, ALA:264, GLN:346, and SER:350. Moreover, hydrophobic interactions were notable for residues such as HIS:266, VAL:312, VAL:345, PHE:314, and PHE:175. A small number of ionic interactions was observed around ARG:176 and TYR:183 (Fig 5C and E). Furthermore, in the SIRT2_MSID001658, the water bridge interactions were robust and persistent, particularly notable with residues such as ALA:164, GLN:246, VAL:312, and ASN:365. When it came to SER:342, ASN:365, and GLU:367, the hydrogen bonding was very noticeable. In addition, hydrophobic interactions were shown to be important for residues such as PHE:314, PHE:198, PHE:345, and PHE:175 (Fig 5D and F).

#### 3.4.6. Principal component analysis (PCA).

**Fig 6:**
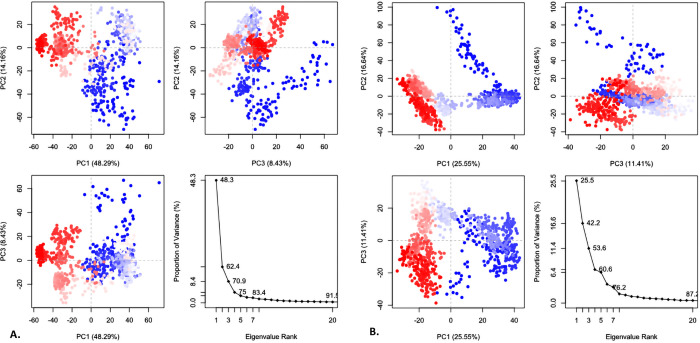
The principal component analysis of the SIRT2_MSID000672 (A) and SIRT2_MSID001658 (B) complexes.

To evaluate the fundamental dynamics and conformational flexibility of protein-ligand complexes during the MD simulations, PCA was performed. The eigenvalue distributions and clustering patterns of two complexes (SIRT2_MSID000672 and SIRT2_MSID001658) were assessed along the first three principal components (PC1, PC2, and PC3). The individual contributions of PC1, PC2, and PC3 to the total variation of SIRT2_MSID000672 were 25.55%, 16.64%, and 11.41%, respectively, accounting for a total variance of approximately 53.6% (Fig 6A). Trajectory projections along PC1 vs. PC2, PC1 vs. PC3, and PC2 vs. PC3 showed clear and dense clustering, especially in the red areas. After the first few components, the eigenvalue scree plot showed a precipitous decline.

The SIRT2_MSID001658, on the other hand, showed that PC1, PC2, and PC3 each contributed 48.29%, 14.16%, and 8.43% of the overall variation, totaling approximately 70.9% (Fig 6B). While PC1 accounted for a larger proportion of the variation than SIRT2_MSID000672, the grouping in the PC projections appeared more scattered and overlapping, especially along the PC1 and PC3 axes. The SIRT2_MSID000672 appeared to have a less flexible and more stable protein-ligand interaction, according to the PCA results, which are supported by its more compact clustering and well-converged conformational ensemble.

#### 3.4.7. Dynamic cross-correlation matrix (DCCM) analysis.

**Fig 7:**
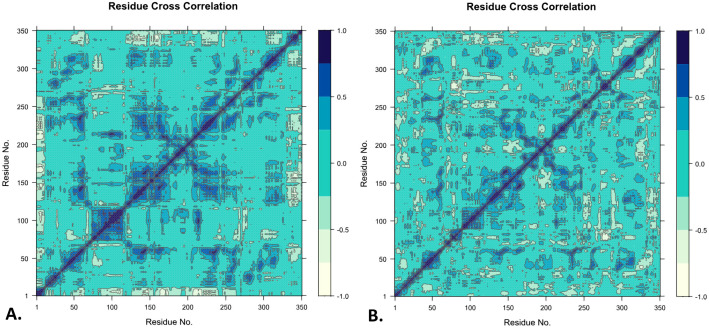
The dynamic cross-correlation matrix (DCCM) analysis the SIRT2_MSID000672 (A) and SIRT2_MSID001658 (B) complexes.

To evaluate the internal motion relationships among the residues of the protein-ligand complexes in the MD simulations, the DCCM analysis was employed. DCCM indicated that the overall correlation ranged from −1.0 to 1.0 (from sea green to dark blue). The diagonal line indicated self-correlation (value = 1) (Fig 7).

The presence of positively correlated motions across the matrix was more common in the SIRT2_MSID000672, especially in many off-diagonal regions. This provides more evidence that the SIRT2_MSID000672 ligand binding stabilizes the protein’s global mobility and facilitates coordinated dynamics of its residues. The SIRT2_MSID000672 overall matrix displayed a more uniformly distributed correlation pattern. The SIRT2_MSID001658, on the other hand, showed less consistent and more dispersed correlation patterns, with specific areas showing weaker or more varied connections than others. With fewer clearly defined interaction domains and more anti-correlated movements, the protein appeared to be more structurally flexible and exhibits less coherent dynamic behavior when bound to ligands (Fig 7). The findings showed that, in terms of dynamic compatibility and potential effectiveness, SIRT2_MSID000672 has a superior protein-ligand interaction.

## 4. Discussion

Recent findings highlight the crucial role of SIRT2 in regulating pathways linked to cancer, particularly those involved in tumor growth and metastasis. The protein, as a member of the NAD-dependent deacetylase family, plays a crucial role in essential cellular processes, including cell cycle progression, genome stability, and metabolic regulation, making it an intriguing target in cancer research. The distinctive configuration of its active site presents an avenue for developing selective inhibitors that hold significant therapeutic promise. In this context, fungal metabolites emerge as a significant source of structurally diverse and biologically active compounds. Utilizing these natural products to produce SIRT2 inhibitors may lead to the development of effective anticancer agents featuring innovative mechanisms of action.

The current study utilized CADD to develop anticancer agents targeting SIRT2, utilizing fungal secondary metabolites obtained from MeFSAT. Subsequent pharmacokinetic studies revealed that all metabolites had significant gastrointestinal absorption, indicating satisfactory oral bioavailability [54,55]. They were impermeable to the blood-brain barrier, suggesting no evidence of neurological or neuroprotective effects [56]. Moreover, none of the substances were recognized as P-gp substrates, thereby diminishing the probability of active efflux and potentially augmenting intracellular drug retention, which could enhance therapeutic effectiveness [55]. All bioactive substances adhered to Lipinski’s Rule of Five without violations, indicating superior oral bioavailability. Moreover, all metabolites satisfied the Ghose, Veber, Egan, and Muegge filter requirements, therefore reinforcing their drug-likeness and pharmacokinetic viability [57]. The bioavailability score for all drugs was 0.55, indicating potential for oral absorption [58].

The findings demonstrated that all fungal metabolites were non-hepatotoxic, indicating a minimal risk of liver toxicity, which is essential for drug metabolism and systemic tolerance [59]. All metabolites were classified as non-carcinogenic, indicating an absence of cancer-related adverse effects [59]. Furthermore, none of the metabolites exhibited mutagenicity or cytotoxicity, hence substantiating their potential safety for therapeutic use. The results suggested that the fungal metabolites exhibited favorable toxicity profiles, making them strong candidates for drug optimization and subsequent preclinical assessment [59,60].

The post-docking analysis of selected fungal metabolites with SIRT2 revealed a spectrum of binding affinities, suggesting varied interaction potentials. Among the nine compounds evaluated, MSID001658 exhibited the highest binding affinity with a docking score of –10.9 kcal/mol, indicating a strong and stable interaction with the SIRT2 active site. This was closely followed by MSID001657 and MSID000672, with scores of –10.5 and –10.2 kcal/mol, respectively, reinforcing their potential as potent SIRT2 inhibitors. MSID001567 and MSID000670 also demonstrated substantial binding strength, suggesting favorable interactions that may translate to effective inhibition [54]. Compounds such as MSID000673, MSID001656, and MSID000671 showed moderate binding affinities (–9.4 to –8.9 kcal/mol), which may still be therapeutically relevant depending on their pharmacokinetic and toxicity profiles. The lowest docking score was recorded for MSID000474 (–8.5 kcal/mol), suggesting comparatively weaker binding and reduced potential as a lead compound. Overall, the docking results highlight MSID001658, MSID001657, and MSID000672 as the most promising candidates for further investigation, given their superior binding energies and presumed stability within the SIRT2 binding pocket [54].

The 100 ns molecular dynamics simulation demonstrated unique stability patterns for SIRT2 complexes with MSID000672 and MSID001658. While both ligands revealed broad stability, MSID000672 exhibited greater RMSD fluctuations, suggesting moments of transient instability at certain intervals [61–63]. On the other hand, MSID001658 exhibited a stable structural integrity throughout. Notably, SIRT2 within the MSID001658 complex exhibited more pronounced dynamic fluctuations compared to the MSID000672 complex, particularly after the 50 ns mark. Despite the mid-simulation events, a slow decrease in RMSD as the simulation ends indicates a movement toward re-equilibration and stabilization, highlighting the likelihood of both ligands to establish relatively stable complexes with SIRT2 as time progresses [61–64]. In the 100 ns simulation, the SIRT2_MSID000672 complex demonstrated increased protein flexibility compared to SIRT2_MSID001658, as indicated by its higher RMSF values. Significant variations were noted in the residue regions 15–36, 66–78, 240–293, and 333–350, suggesting increased local mobility and possible conformational flexibility in the SIRT2_MSID000672 complex [61–63].

During the 100 ns simulation, the complexes of SIRT2 with MSID000672 and MSID001658 exhibited unique structural dynamics. SIRT2_MSID000672 exhibited elevated SASA and PSA values, indicating increased surface exposure and polarity. On the other hand, SIRT2_MSID001658 showed a marginally increased MolSA and a more compact Rg, suggesting tighter packing. The number of hydrogen bonds observed in the complexes showed a similar pattern, indicating a stable interaction throughout. In summary, the findings indicate that MSID001658 creates a more compact and stable association with SIRT2, whereas MSID000672 provides enhanced structural flexibility and solvent accessibility.

The interaction dynamics of SIRT2 with the MSID000672 and MSID001658 revealed distinct binding profiles. The MSID000672 engaged HIS:266 and GLN:246 via hydrogen bonds and hydrophobic contacts, with robust water bridge interactions involving residues like PHE:175 and GLN:346. In contrast, the MSID001658 exhibited diverse functional groups and formed key water-mediated and hydrophobic interactions with ALA:164, VAL:312, and PHE:198. Bar graph analysis highlighted consistent hydrogen bonds, hydrophobic contacts, and water bridges across both complexes. Notably, the MSID001658 showed stronger water bridge persistence, suggesting enhanced solvation dynamics, while MSID000672 exhibited greater direct hydrogen bonding, supporting stable ligand anchoring.

The PCA analysis showed that the SIRT2_MSID000672 displayed tighter clustering and represented 53.6% of the total variance, suggesting enhanced conformational stability. Conversely, the SIRT2_MSID001658 exhibited a variance of 70.9% with a more scattered clustering pattern, indicating greater flexibility. The findings suggest that the MSID000672 creates a more stable and compact complex with SIRT2 throughout the simulation period. The DCCM analysis showed that the SIRT2_MSID000672 displayed more robust and extensive positive correlations among residues, suggesting stable and coordinated internal movements. The correlation matrix, which is uniformly distributed, indicates improved structural integrity when ligands bind. In comparison, the SIRT2_MSID001658 exhibited more dispersed and less robust correlations, indicating increased flexibility and a lack of cohesive dynamics. The results indicated that the SIRT2_MSID000672 provide a more stable and dynamically compatible complex, which may enhance interaction efficiency.

## 5. Conclusion

This research highlights the promising therapeutic potential of targeting SIRT2 in cancer treatment by utilizing fungal secondary metabolites as novel inhibitors. Through computational approaches, several promising fungal secondary metabolites were identified from the MeFSAT database, with MSID001658 and MSID000672 standing out as the leading candidates due to their robust binding affinities, favorable pharmacokinetics, and safe profiles. The molecular dynamics simulation analysis uncovered unique interaction patterns: the MSID001658 established a compact and stable complex, whereas the MSID000672 provided increased flexibility and coordination within the SIRT2 binding pocket. These findings collectively underscore the potential for developing effective, safe, and orally bioavailable SIRT2-targeted anticancer agents derived from fungal metabolites, setting the stage for further preclinical validation and drug refinement.

## Supporting information

S1 Table The bioactive compounds of the fungal metabolites with their MeFSAT identifiers, PubChem ID, metabolite name, chemical formula, SMILES, and chemical structures. (DOCX)

S2 Table Biophysical properties of the fungal metabolites predicted by SwissADME. (DOCX)

S3 Table Pharmacokinetics properties of the fungal metabolites predicted by SwissADME. (DOCX)

S4 Table Drug-likeness and bioavailability of the fungal metabolites by SwissADME. (DOCX)

S5 Table Toxicity analysis of the fungal metabolites by ProTox 3.0. (DOCX)

S1 Fig The post-docking analysis of the standard inhibitors of SIRT2, including sirreal (A) and AGK2 (B). (TIF)

S2 Fig The post-simulation analysis of SIRT2 (Cα protein), including RMSF (A) and RMSF (B) analysis. (TIF)
