# Supplementary material for: Bioactive fungal metabolites as SIRT2 antagonists: A computational quest for cancer treatment
Source: PLoS One. 2025 Dec 22;20(12):e0339474. doi: 10.1371/journal.pone.0339474 (PMC12721511; doi:10.1371/journal.pone.0339474)
Supplement: S1 Table — (DOCX) [file pone.0339474.s001.docx]

**Table S1.** The bioactive compounds of the fungal metabolites with their MeFSAT identifiers, PubChem ID, metabolite name, chemical formula, SMILES, and chemical structures.

| MeFSAT  identifier | Metabolite chemical name | Chemical formula | SMILES | Chemical structure |
| --- | --- | --- | --- | --- |
| MSID001658 | **Caputmedusin K** | C19H21NO7 | OC(=O)CCCN1Cc2c(C1=O)cc1c(c2O)CC(O1)C1(C)CCC(=O)O1 | 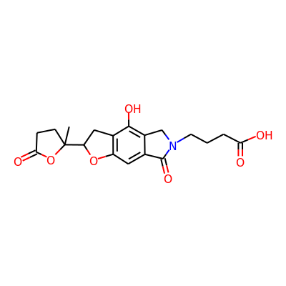 |
| MSID001657 | Caputmedusin j | C19H21NO7 | OC(=O)CCCN1Cc2c(C1=O)cc1c(c2O)CC(O1)C1(C)CCC(=O)O1 | 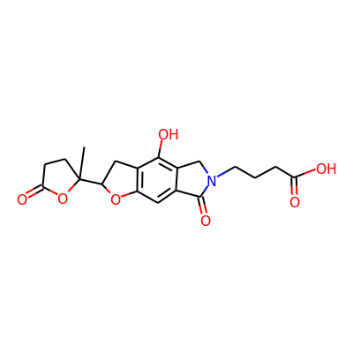 |
| MSID000672 | Erinacerin S | C18H19NO6 | C/C(=CCc1c(O)cc2c(c1O)C(=O)NC2=O)/CC/C=C(/C(=O)O)C | 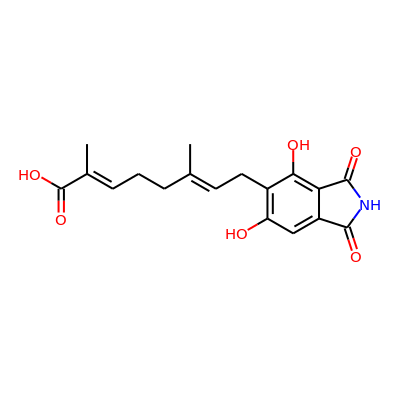 |
| MSID001567 | Caputmedusin I | C20H25NO7 | COC(=O)CCCN1Cc2c(C1=O)cc(c(c2O)C/C=C(/CCC(=O)O)C)O | 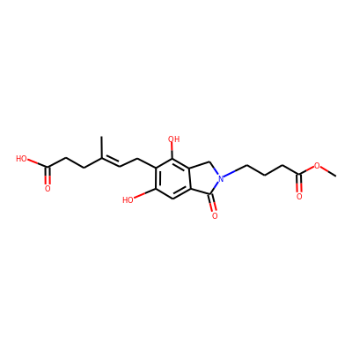 |
| MSID000670 | Erinacerin Q | C26H29NO5 | C/C(=CCc1c(O)cc2c(c1O)CN(C2=O)CCc1ccccc1)/CC/C=C(/C(=O)O)C | 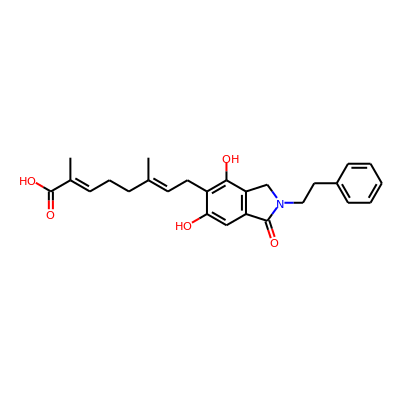 |
| MSID000673 | Erinacerin T | C16H17NO6 | COC(=O)CC/C(=C/Cc1c(O)cc2c(c1O)C(=O)NC2=O)/C | 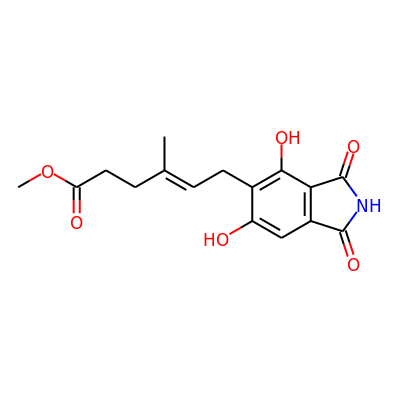 |
| MSID001656 | Caputmedusin H | C20H25NO7 | COC(=O)CC/C(=C/Cc1c(O)cc2c(c1O)CN(C2=O)CCCC(=O)O)/C | 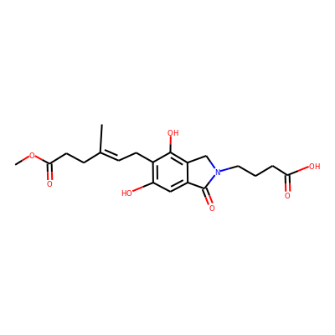 |
| MSID000671 | Erinacerin R | C17H21NO5 | CCOC(=O)CC/C(=C/Cc1c(O)cc2c(c1O)CNC2=O)/C | 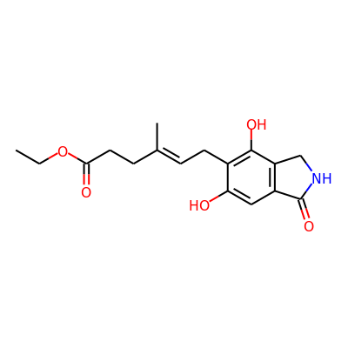 |
| MSID000474 | Catechin | C15H14O6 | Oc1cc2O[C@H](c3ccc(c(c3)O)O)[C@H](Cc2c(c1)O)O | 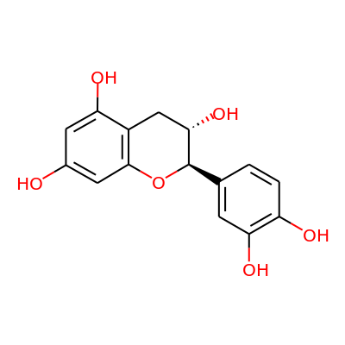 |
