# Supplementary material for: Bioactive fungal metabolites as SIRT2 antagonists: A computational quest for cancer treatment
Source: PLoS One. 2025 Dec 22;20(12):e0339474. doi: 10.1371/journal.pone.0339474 (PMC12721511; doi:10.1371/journal.pone.0339474)
Supplement: S2 Table — (DOCX) [file pone.0339474.s002.docx]

**Table S2.** Biophysical properties of the fungal metabolites predicted by SwissADME.

| Fungal metabolite | Biophysical properties | | | | | | |
| --- | --- | --- | --- | --- | --- | --- | --- |
|  | Molecular weight (g/mol) | Heavy atoms | Aromatic heavy atoms | Fraction Csp3 | Molar refractivity | Rotatable bonds | TPSA |
| MSID001658 | 375.37 | 27 | 6 | 0.53 | 97.1 | 5 | 113.37 |
| MSID001657 | 375.37 | 27 | 6 | 0.53 | 97.1 | 5 | 113.37 |
| MSID000672 | 345.35 | 25 | 6 | 0.28 | 94.92 | 6 | 123.93 |
| MSID001567 | 391.42 | 28 | 6 | 0.45 | 105.96 | 10 | 124.37 |
| MSID000670 | 435.51 | 32 | 12 | 0.31 | 128.69 | 9 | 98.07 |
| MSID000673 | 319.31 | 23 | 6 | 0.31 | 85.29 | 6 | 112.93 |
| MSID001656 | 391.42 | 28 | 6 | 0.45 | 105.96 | 10 | 124.37 |
| MSID000671 | 319.35 | 23 | 6 | 0.41 | 89.68 | 7 | 95.86 |
| MSID000474 | 290.27 | 21 | 12 | 0.2 | 74.33 | 1 | 110.38 |
