# Supplementary material for: Bioactive fungal metabolites as SIRT2 antagonists: A computational quest for cancer treatment
Source: PLoS One. 2025 Dec 22;20(12):e0339474. doi: 10.1371/journal.pone.0339474 (PMC12721511; doi:10.1371/journal.pone.0339474)
Supplement: S3 Table — (DOCX) [file pone.0339474.s003.docx]

**Table S3.** Pharmacokinetics properties of the fungal metabolites predicted by SwissADME

| Fungal metabolite | Pharmacokinetics Properties | | | | | | |  |  |
| --- | --- | --- | --- | --- | --- | --- | --- | --- | --- |
|  | GI absorption | **BBB permeant** | **P-gp substrate** | **CYP1A2 inhibitor** | **CYP2C19 inhibitor** | CYPC9 inhibitor | **CYP2D6 inhibitor** | **CYP3A4 inhibotor** | **Log Kp**  **(**cm/s) |
| MSID001658 | High | No | Yes | No | No | No | No | No | -8.07 |
| MSID001657 | High | No | Yes | No | No | No | No | No | -8.07 |
| MSID000672 | High | No | No | Yes | No | No | No | No | -6.25 |
| MSID001567 | High | No | Yes | Yes | No | No | No | No | -7.45 |
| MSID000670 | High | No | No | No | No | Yes | No | No | -5.63 |
| MSID000673 | High | No | No | Yes | No | No | No | No | -6.69 |
| MSID001656 | High | No | Yes | Yes | No | No | No | No | -7.45 |
| MSID000671 | High | No | Yes | Yes | No | No | No | No | -6.78 |
| MSID000474 | High | No | Yes | No | No | No | No | No | -7.82 |
