# Supplementary material for: Bioactive fungal metabolites as SIRT2 antagonists: A computational quest for cancer treatment
Source: PLoS One. 2025 Dec 22;20(12):e0339474. doi: 10.1371/journal.pone.0339474 (PMC12721511; doi:10.1371/journal.pone.0339474)
Supplement: S4 Table — (DOCX) [file pone.0339474.s004.docx]

**Table S4.** Drug-likeness and bioavailability of the fungal metabolites by SwissADME.

| Fungal metabolite | Drug likeness properties | | | | | |
| --- | --- | --- | --- | --- | --- | --- |
|  | Lipinski, Violation | Ghose | Veber | Egan | Muegge | Bioavailability score |
| MSID001658 | Yes; 0 violation | Yes | Yes | Yes | Yes | 0.56 |
| MSID001657 | Yes; 0 violation | Yes | Yes | Yes | Yes | 0.56 |
| MSID000672 | Yes; 0 violation | Yes | Yes | Yes | Yes | 0.56 |
| MSID001567 | Yes; 0 violation | Yes | Yes | Yes | Yes | 0.56 |
| MSID000670 | Yes; 0 violation | Yes | Yes | Yes | Yes | 0.56 |
| MSID000673 | Yes; 0 violation | Yes | Yes | Yes | Yes | 0.55 |
| MSID001656 | Yes; 0 violation | Yes | Yes | Yes | Yes | 0.56 |
| MSID000671 | Yes; 0 violation | Yes | Yes | Yes | Yes | 0.55 |
| MSID000474 | Yes; 0 violation | Yes | Yes | Yes | Yes | 0.55 |
