# Supplementary material for: Bioactive fungal metabolites as SIRT2 antagonists: A computational quest for cancer treatment
Source: PLoS One. 2025 Dec 22;20(12):e0339474. doi: 10.1371/journal.pone.0339474 (PMC12721511; doi:10.1371/journal.pone.0339474)
Supplement: S5 Table — (DOCX) [file pone.0339474.s005.docx]

**Table S5.** Toxicity analysis of the fungal metabolites by ProTox 3.0.

| Fungal metabolite | Toxicity | | | | | |
| --- | --- | --- | --- | --- | --- | --- |
|  | Hepatotoxicity | Carcinogenicity | Mutagenicity | Cytotoxicity | **Cardiotoxicity** | **Nephrotoxicity** |
| MSID001658 | Inactive | Inactive | Inactive | Inactive | Inactive | Inactive |
| MSID001657 | Inactive | Inactive | Active | Inactive | Inactive | Inactive |
| MSID000672 | Inactive | Inactive | Inactive | Inactive | Inactive | Inactive |
| MSID001567 | Inactive | Inactive | Inactive | Inactive | Inactive | Inactive |
| MSID000670 | Inactive | Inactive | Inactive | Inactive | Inactive | Inactive |
| MSID000673 | Inactive | Inactive | Inactive | Inactive | Inactive | Inactive |
| MSID001656 | Inactive | Inactive | Inactive | Inactive | Inactive | Inactive |
| MSID000671 | Inactive | Inactive | Inactive | Inactive | Inactive | Inactive |
| MSID000474 | Inactive | Inactive | Inactive | Inactive | Inactive | Inactive |
