# Supplementary figures and images for: Bioactive fungal metabolites as SIRT2 antagonists: A computational quest for cancer treatment
Source: PLoS One. 2025 Dec 22;20(12):e0339474. doi: 10.1371/journal.pone.0339474 (PMC12721511; doi:10.1371/journal.pone.0339474)

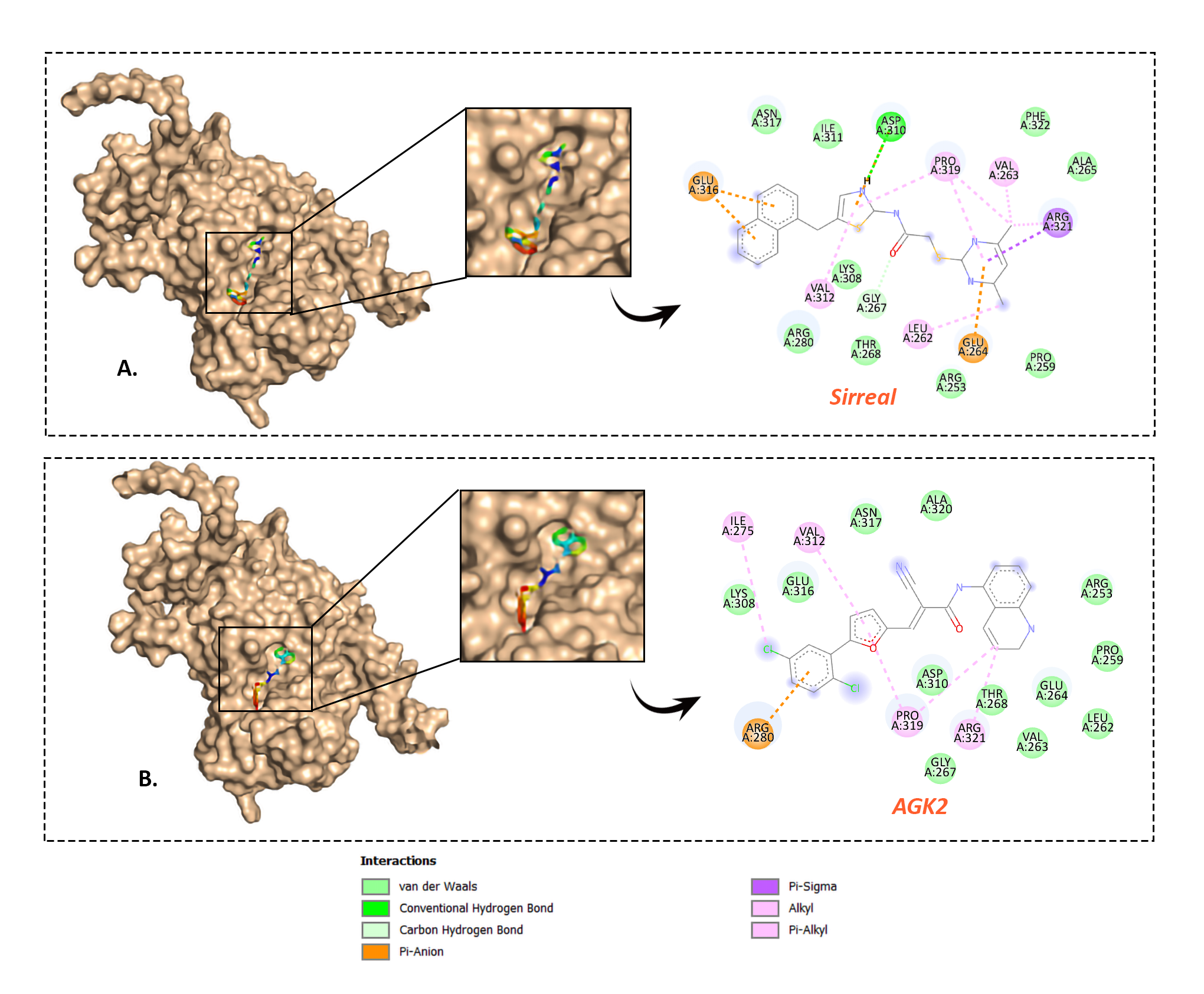

Supplement: S1 Fig — (TIF) [file pone.0339474.s006.tif]

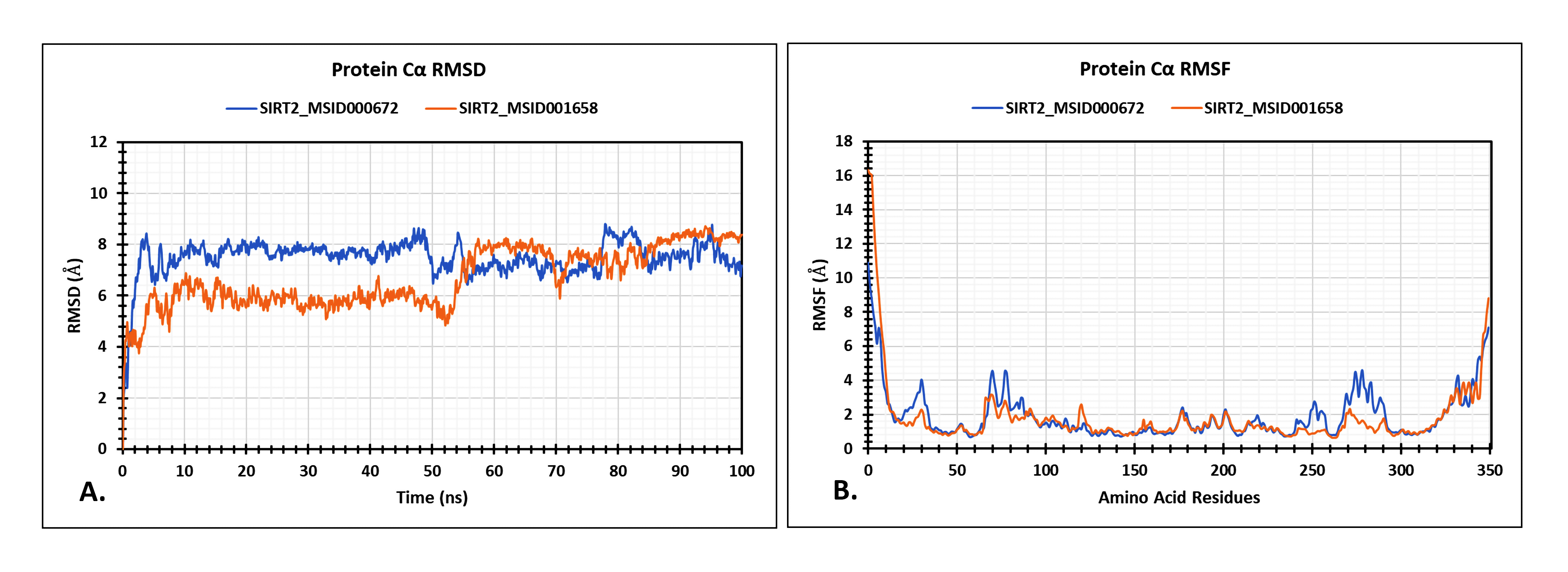

Supplement: S2 Fig — (TIF) [file pone.0339474.s007.tif]
